# Editorial: Machine learning approaches to recognize human emotions

**DOI:** 10.3389/fpsyg.2023.1333794

**Published:** 2024-01-04

**Authors:** Camilo E. Valderrama, Marcelo Gitirana Gomes Ferreira, Juan Manuel Mayor Torres, Alejandro Rafael Garcia-Ramirez, Sergio G. Camorlinga

**Affiliations:** ^1^Department of Applied Computer Science, University of Winnipeg, Winnipeg, MB, Canada; ^2^Department of Design, Santa Catarina State University, Florianopolis, Brazil; ^3^Department of Information Engineering and Computer Science, University of Trento, Trento, Italy; ^4^Digital Ware Advisory Inc., Bogota, Colombia; ^5^Department of Computer Engineering, Universidade do Vale de Itajai, Itajai, Brazil

**Keywords:** emotion recognition, machine learning, deep learning, facial expressions, sentiment analysis, EEG, wearable devices, biosignals

Emotion recognition is critical in human interaction, as it guides individuals in responding appropriately to others' feelings (Dzedzickis et al., 2020; Li et al., 2021). Unfortunately, individuals diagnosed with neurodevelopmental disorders often struggle to perceive and understand emotions, thus limiting their interaction with others (Livingston and Happé, 2017). One solution to assist these individuals is to leverage the current rise of Artificial Intelligence (AI) to develop data-driven methods capable of predicting emotions from different sources, such as brain and peripheral biosignals, facial gestures, speech, text, and many others (Bota et al., 2019).

In this Research Topic, we have addressed this direction by presenting seven high-quality manuscripts that applied AI and Machine Learning (ML) to recognize emotions from physiological signals, images, or text. Our issue differs from other contemporary emotion recognition-related literature by including papers attempting to recognize emotion from different sources, thus approaching the common objective of predicting emotions from different perspectives. A summary of the research papers published on this topic is provided below, categorizing them into five main sections.

## Biosignal processing for emotion recognition

One of the most promising sources for emotion recognition is biosignals (Egger et al., 2019). Sajno et al. conducted a comprehensive review of the potential of biosignals for emotion recognition. They underscored the capacity of ML systems to extract relevant features from biosignals, such as electroencephalogram (EEG) and electrocardiogram (ECG), to recognize emotion. This capacity enables the development of applications for diagnostic precision medicine, affective computing, and brain-computer interfaces. The authors also discussed the potential benefits and limitations, emphasizing the crucial requirement for interdisciplinary collaboration between mental health professionals and data scientists to ensure the success of machine learning-based applications in the mental health domain.

## Facial expressions to improve self-criticism detection

Self-criticism is a cognitive process wherein individuals engage in reflection and self-evaluation of emotion and cognition ratings. However, when this process becomes uncontrolled, particularly in cases of depression, it can lead to self-harm, self-loathing, or self-aggression (Kannan and Levitt, 2013). Current methods for assessing this condition rely on questionnaires, which can be problematic due to potential response bias. To offer a more objective measurement of self-criticism, Halamová et al. analyzed the facial expressions displayed by 80 individuals while engaging in self-criticism using the two-chair technique. The analysis revealed that the most frequently observed facial expressions during self-criticism were dimpling, lip pressing, eye closure, jaw dropping, and outer brow raising. These expressions can thus serve as objective indicators for diagnosing excessive self-criticism.

## Chatbot for text emotion recognition

Emotions are conveyed in all forms of communication, including written communication (Alswaidan and Menai, 2020). This is especially evident in contemporary life, where individuals use chat applications to interact with both humans and artificial intelligent entities. Machová et al. explored this avenue by developing a chatbot to provide a companion to older adults. The chatbot extracted semantic features from user-provided text and then used a deep model comprising a Convolutional Neural Network (CNN) and a Long Short-Term Memory (LSTM) network to predict the user's emotions. Based on the predicted emotion, the chatbot selected an appropriate pre-prepared response to continue the conversation with the user. Their approach illustrates the promise of AI-based emotion recognition in improving human-machine interactions.

Elyoseph et al. also explored the capabilities of chatbots for emotion recognition by assessing ChatGPT's ability to identify the emotions elicited by 20 scenarios from the Level of Emotional Awareness Scale (LEAS) (Lane et al., 1990). Two psychologists assessed ChatGPT's responses, comparing its performance in detecting emotional awareness (EA) with that of 750 humans (Nandrino et al., 2013). The comparison revealed that ChatGPT can identify EA more accurately than the general population. This application underscores the benefits that ChatGPT can offer to individuals with EA impairments.

## EEG-based emotion recognition

EEG signals have emerged as the preferred method for emotion detection due to their objective nature, as opposed to methods that individuals can manipulate, such as facial expressions, body posture, or speech (Wang and Wang, 2021). In our research, Masuda and Yairi used EEG and eight other peripheral signals to recognize fear across four intensity levels. They emphasized the advantages of employing deep learning models, such as CNN and LSTM, for emotion recognition, as these deep decoders can automatically extract features from EEG signals. Their CNN+LSTM model was tested on the DEAP dataset (Koelstra et al., 2011), achieving an impressive emotion-recognition accuracy of 98.79%.

Similarly, Mouri et al. aimed to identify which EEG channel pairs and frequency bands were more significant for predicting emotions. To achieve this, the authors measured different brain-signal asymmetries by pairing equidistant EEG channels and calculating energy ratios for the δ (1–4 Hz), θ (4–8 Hz), α (8–12 Hz), β (12–30 Hz), and γ (30–50 Hz) bands. These energy-based features were used to train four binary logistic regressions for predicting the emotions of 15 healthy individuals included in the SEED-IV dataset (Zheng et al., 2018). The authors found that the energy ratios between lateral frontal, parietal, and temporal EEG pairs (FT7-FT8, T7-T8, and TP7-TP8) were the most relevant for predicting emotions, particularly in the α and γ bands. This study demonstrates the feasibility of using brain-signal asymmetry measures, especially energy ratios, in emotion recognition.

## Wearable sensor for detecting obsessive compulsive disorder events

As the use of wearable devices, such as smartwatches, becomes increasingly common, Lønfeldt et al. investigated how wrist-worn biosensors and machine learning (ML) can be used to detect obsessive-compulsive disorder (OCD) episodes in eight adolescents. Over 8 weeks, the participants wore the biosensors, which recorded their physiological data, including Blood Volume Pulse (BVP), Skin Temperature (ST), and Electrodermal Activity (EA). Participants also documented experienced OCD events during their daily activities. The collected data was used to train various ML models, resulting in a maximum accuracy of 70%. This research suggests the potential use of wearable biosensors for detecting OCD episodes.

## Concluding remarks

AI state-of-the-art has provided technology that enables the development of systems that can recognize emotion from different data types, such as text from chatbots, neural signals from brain-machine interfaces, facial expressions from videos and pictures, and peripheral physiological signals from wireless wearable devices. These systems hold the potential for aiding individuals with neurodevelopmental disorders in managing conditions such as anxiety, depression, and bipolar disorder, thereby enhancing their quality of life.

## Author contributions

CV: Conceptualization, Writing—original draft, Writing—review & editing. MG: Writing—original draft, Writing—review & editing. JM: Writing—review & editing. AG-R: Writing—review & editing. SC: Writing—review & editing.
